# The effectiveness of virtual reality in enhancing mindfulness: a systematic review and meta-analysis

**DOI:** 10.3389/fpubh.2025.1709782

**Published:** 2025-11-17

**Authors:** Fen Xie, Huanjie Zheng, Xiaojuan Hu

**Affiliations:** 1 School of Journalism and Communication, Guangxi Normal University, Guilin, Guangxi, China; 2 Faculty of Education, Guangxi Normal University, Guilin, Guangxi, China

**Keywords:** virtual reality, mindfulness, digital health, meta-analysis, health communication

## Abstract

**Background:**

In recent years, virtual reality (VR) has been increasingly applied in mindfulness training. Although the number of studies in this area has grown rapidly, their quality remains inconsistent. Substantial variations exist across studies in terms of participant populations, modes of VR application, experimental designs, intervention durations, and measurement instruments, which in turn have led to divergent findings. Therefore, a systematic review and meta-analysis is warranted to synthesize the existing evidence, evaluate the robustness of current findings, and provide guidance for future research and clinical applications.

**Methods:**

A meta-analytic approach was employed, incorporating 25 studies published in both Chinese and English with a total sample of 1,485 participants. A random-effects model was applied, and Hedges’ g was used as the effect size metric to evaluate the effectiveness of VR in enhancing mindfulness. The study also examined the potential moderating effects of participant characteristics, modes of VR implementation, experimental designs, intervention durations, and measurement instruments.

**Results:**

(1) VR interventions significantly enhanced mindfulness, with a large effect size (Hedges’ g = 0.975). (2) The effectiveness of VR-based mindfulness training was moderated by usage mode and participants’ health status. Fully immersive VR demonstrated greater benefits than active-interaction VR. Participants with mental health disorders experienced greater improvements compared to healthy individuals, whereas those with chronic physical conditions showed no significant effects. (3) No significant moderating effects were observed for experimental design, intervention duration, or measurement instruments.

**Discussion:**

The findings of this study contribute to the theoretical foundation of mindfulness training and offer practical implications for the design of future VR-based interventions. Specifically, the results suggest that prioritizing the development of highly immersive, low-interaction, and nature-friendly virtual environments may enhance the effectiveness of VR interventions.

## 1 Introduction

Mindfulness originated from Buddhist meditation traditions, with its core principle being awareness of the present moment, which emphasizes attending to current experiences with a nonjudgmental and open attitude (1). Awareness and openness are considered two fundamental characteristics of mindfulness (2). Since its introduction into psychology in the late 1970s, mindfulness has gradually been integrated into clinical practice. By the 1980s, it had been adopted as a therapeutic approach in medicine, leading to the development of structured interventions such as Mindfulness-Based Stress Reduction (MBSR) and Mindfulness-Based Cognitive Therapy (MBCT) (3). With the increasing pressures of modern life, mindfulness has attracted growing scholarly attention as an effective strategy for stress reduction (4). Mindfulness-based interventions encourage individuals to focus on their present state with a calm, open, and accepting attitude (5). A substantial body of research has demonstrated that mindfulness interventions exert positive effects across a wide range of domains, including anxiety, depression, chronic pain, insomnia, and stress management (6, 7). Nevertheless, traditional approaches to mindfulness training often pose considerable challenges for many individuals. Practices such as seated meditation demand sustained concentration and repeated exercises, which beginners may find difficult to maintain due to monotony, environmental distractions, or lack of motivation (8). In response, researchers have increasingly explored how emerging technologies might better support mindfulness practice (7, 9), particularly through the use of visual and symbolic representations designed to enhance engagement and sustain interest (10).

Among emerging technologies, VR, particularly immersive VR, has attracted considerable attention. VR is defined as a computer-generated environment that allows users to experience a strong sense of presence in simulated spaces distinct from their physical surroundings (11). Empirical evidence suggests that VR-based mindfulness interventions can effectively alleviate anxiety and stress (12). With the rapid growth of research in this field, however, the existing literature demonstrates substantial methodological heterogeneity and uneven study quality. Participants range from non-clinical populations such as university students and corporate employees to clinical groups including individuals with schizophrenia, brain injury, and anxiety or depression. Research designs vary widely, encompassing randomized controlled trials (RCTs) as well as within-subject pre-post comparisons. Intervention duration ranges from a single 20-min session to structured eight-week programs, while outcome measures include diverse instruments such as the MAAS, TMS, and FFMQ. This heterogeneity has contributed to inconsistent findings. While many studies report that VR significantly enhances mindfulness, the magnitude of effects varies, and some studies have suggested only limited benefits (13). As some scholars have noted, our understanding of how VR can facilitate the acquisition of mindfulness skills remains incomplete (14). A central scientific question arises: to what extent can VR enhance mindfulness, and which factors influence the magnitude of its effects? A significant gap exists in the literature regarding the systematic evaluation of VR interventions’ effects on mindfulness. Key factors, including usage patterns, participant health status, intervention duration, assessment instruments, and experimental design, must be elucidated to advance our comprehensive understanding of their moderating effects. In response, there is an urgent need in the academic community to systematically and quantitatively synthesize the existing evidence using meta-analytic methods (15). Meta-analysis, as a literature-based quantitative approach, enables the integration of effect sizes across multiple studies to estimate a pooled effect, thereby providing more reliable and generalizable conclusions than individual studies alone (16).

Therefore, the present study aims to conduct a systematic review and meta-analysis to comprehensively evaluate the effectiveness of VR in enhancing mindfulness. Furthermore, it examines the potential moderating effects of factors such as VR usage mode, participants’ health status, study design, intervention duration, and measurement instruments. This study seeks to provide robust empirical evidence to inform both theoretical research and practical applications.

## 2 Literature review

In addition to exploring the overall effect of VR on enhancing mindfulness levels, this study will also analyze the moderating variables that influence the relationship between the two. Based on the data obtained from the literature review and coding, the study primarily examines the moderating effects of usage mode, participants’ health status, study design, intervention duration, and measurement tools.

### 2.1 The effects of virtual reality on mindfulness

Mindfulness practices take various forms, including seated meditation, dynamic meditation, and breathing exercises (17, 18). Regular engagement in mindfulness meditation has been shown to provide numerous psychological benefits, such as enhancing a sense of calm, reducing stress, alleviating symptoms of depression and anxiety, and improving cognitive control (19, 20). Digital media can offer personalized guidance for individual mindfulness training (21). With the advancement of digital technologies, researchers have increasingly integrated VR with mental health interventions to leverage technological advantages in addressing stress-related health challenges. Evidence suggests that VR can significantly enhance mindfulness meditation and support the maintenance of inner calm (7). Virtual reality (VR) is a computer-generated simulation of real-world environments. When users immerse themselves in these virtual sensory environments, their attentional focus on sensory experiences is significantly enhanced, external distractions are reduced, and a sense of presence comparable to real-world experiences is achieved (22). Compared with traditional mindfulness practices such as meditation or seated Zen exercises, VR can lower the difficulty of practice, particularly for individuals with limited time availability (23). Overall, VR-based mindfulness interventions have been shown to effectively enhance positive affect (24), reduce negative emotions such as depression (25), and improve sleep quality (26). Neuroscientific evidence further indicates that participants engaging in VR-guided meditation exhibit overall reductions in *β*-wave activity, suggesting that VR can alleviate anxiety and hyperarousal (15). Although numerous studies have confirmed the effectiveness of VR in enhancing mindfulness, some participants have reported drawbacks such as heavy headsets and insufficient realism of virtual environments. These limitations may reduce user comfort and hinder sustained attentional focus, potentially undermining mindfulness practice (23). Moreover, existing studies vary considerably in terms of participant populations, intervention durations, experimental designs, and outcome measures. Given this heterogeneity, the present study seeks to employ meta-analytic methods to quantitatively evaluate the overall effect of VR on mindfulness.

### 2.2 Moderating variables

Usage Mode. In VR-based mindfulness training, two common usage modes are active-interaction and fully immersion. Participant engagement differs between these modes. In the active-interaction mode, VR systems provide controllers or other input devices that allow users to actively manipulate the environment. Participants exercise greater agency, such as selecting or switching music, visuals, or virtual scenes, while head-mounted display (HMD) sensors record head movements as behavioral data. In contrast, the fully immersive mode does not involve gesture controls or manual operation; participants engage in mindfulness exercises simply by observing and listening to the virtual environment, such as natural scenes or guided audio instructions. Some studies suggest that, compared with active manipulation, fully immersive VR is more conducive to present-moment awareness (27). Conversely, overly complex environments or excessive interaction demands may conflict with the goals of mindfulness training (22). Based on these considerations, active-interaction and fully immersive usage modes may differentially affect attentional allocation, emotion regulation, and behavioral engagement during mindfulness practice, thereby influencing the effectiveness of VR in enhancing mindfulness. Accordingly, we propose the following hypothesis:

*Hypothesis* 1: The effect of VR on mindfulness differs significantly depending on the usage mode.

Health Status. The effectiveness of VR-based mindfulness interventions often depends on participants’ health status, as individuals with different conditions may have distinct objectives and outcomes when engaging with digital interventions (28). Clinical populations experiencing severe anxiety, high stress, or depression often exhibit deficits in attentional control, difficulties in emotion regulation, and recurrent negative affect. For these individuals, the primary benefit of mindfulness training is to help break cycles of negative thinking and emotional relapse (29). In contrast, for patients with chronic illnesses, mindfulness training primarily aims to facilitate disease management, enhance psychological resilience, and mitigate the negative impacts associated with their condition (30). For healthy individuals, the core purpose of mindfulness training is to improve quality of life by fostering present-moment awareness, emotional regulation, and overall well-being (31). Given that VR-based mindfulness interventions offer highly customizable audiovisual environments and immersive experiences, they may produce differential benefits across groups with varying health statuses. Accordingly, we propose the following hypothesis:

*Hypothesis* 2: The effect of VR on mindfulness differs significantly depending on participants’ health status.

Study Design. RCTs are commonly employed in mindfulness intervention research, where participants are randomly assigned to either an intervention or a control group. Randomization maximizes the likelihood of controlling for potentially unobserved confounding variables and, in theory, minimizes their influence on outcomes (32). However, in practice, RCTs are not always feasible, and researchers may rely on observational or quasi-experimental designs to evaluate intervention effects. Compared with randomized experiments, non-randomized designs carry a potential risk of selection bias, as participants may differ in motivation or baseline psychological health, which can influence study outcomes. Overall, randomized experiments are considered more rigorous than non-randomized designs (33). Based on these considerations, we hypothesize that study design may affect the outcomes of mindfulness interventions. Accordingly, we propose the following hypothesis:

*Hypothesis* 3: The effectiveness of VR in enhancing mindfulness levels will differ significantly across experimental conditions.

Intervention Duration. Studies indicate that VR interventions vary in duration, ranging from single-session interventions to multi-session programs. Among multi-session interventions, some last 6 weeks (34), while others extend to 8 weeks (35). Research on traditional mindfulness interventions suggests that the length of the intervention significantly affects participant adherence and engagement, with shorter interventions generally yielding higher completion rates and a greater participant enthusiasm (36). Moreover, intervention duration may influence the magnitude of treatment effects. A meta-analysis focusing on athletes found that mindfulness interventions of different durations produced significantly different effect sizes (37). Although prior research highlights the close relationship between intervention duration and the effectiveness of traditional mindfulness training, this issue has been underexplored in the context of VR-mediated interventions. In virtual reality environments, intervention duration may interact with technological features, such as presence and interactivity, further affecting the effectiveness of VR training and user adherence. Based on this evidence, we propose the following hypothesis:

*Hypothesis* 4: The effect of VR on mindfulness differs significantly depending on intervention duration.

Measurement Tools. Mindfulness is a concept with multiple meanings. Some researchers regard mindfulness as a concrete practice, whereas others define it as a psychological state or a dispositional trait (38, 39). Researchers have developed multiple measurement tools from different perspectives, such as FFMQ, MAAS, SMS, etc. Each scale emphasizes distinct aspects of mindfulness. The FFMQ assesses multidimensional trait mindfulness, highlighting long-term and stable dispositions rather than momentary states. It evaluates both attention to external experiences and awareness and acceptance of internal mental activities, making it suitable for comparing trait mindfulness across different populations (40). The MAAS measures unidimensional trait mindfulness with a focus on attentional awareness in daily life. It is widely used to evaluate mindfulness levels in general populations (1). The SMS, on the other hand, is designed to assess state mindfulness—mindfulness experienced at a particular moment or in specific contexts—rather than enduring traits. This scale is particularly useful for examining short-term changes in mindfulness following interventions (41). In addition to these instruments, other scales have been developed, such as the Applied Mindfulness Process Scale (AMPS), the Athlete Mindfulness Questionnaire (AMQ), and the Comprehensive Inventory of Mindfulness Experiences (CHIME). Commonly used mindfulness measures can be categorized into two groups: (a) general-purpose instruments, such as the FFMQ and MAAS, and (b) context-specific instruments, such as the SMS, CHIME, AMPS, and AMQ (38). These tools are applied in different settings and populations. Given the variability in how mindfulness is conceptualized and measured across studies, the observed effects of VR on mindfulness may be influenced by the choice of measurement instrument. Building on this reasoning, the following research hypothesis is proposed:

*Hypothesis* 5: The effect of VR on mindfulness differs significantly depending on the measurement instrument used.

## 3 Method

### 3.1 Literature screening process

**Figure 1:**
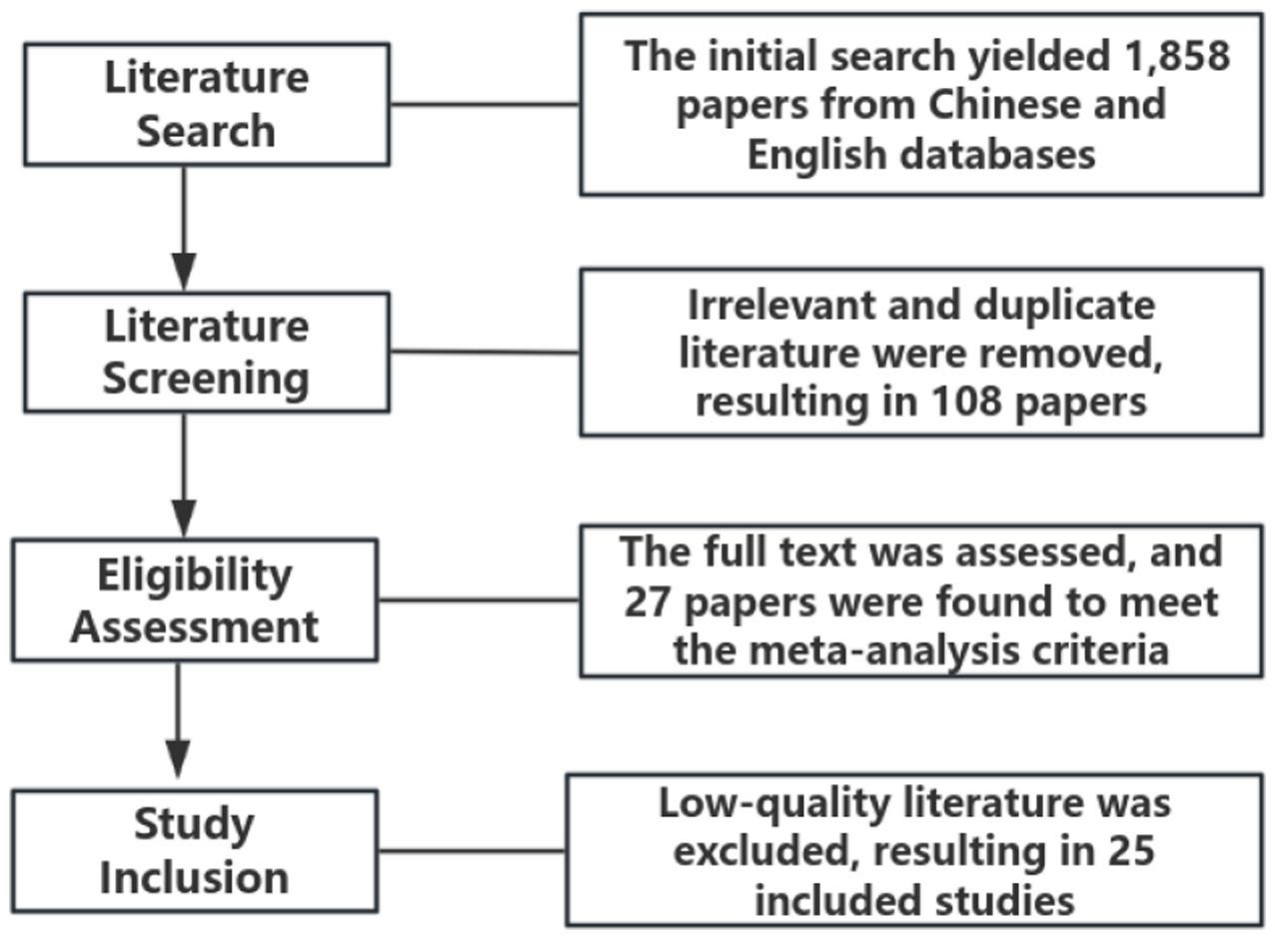
Literature screening flowchart.

This study strictly adhered to the guidelines of the PRISMA Statement for conducting meta-analyses (42). The literature screening was carried out in four stages: search, screening, eligibility assessment, and inclusion (as shown in Figure 1).

Step 1: Literature Search.

The literature search was conducted using both English-and Chinese-language databases. The English databases included Scopus, Web of Science, Cochrane Library, and PubMed; the Chinese databases included China National Knowledge Infrastructure (CNKI), Wanfang Data, and VIP Database. Boolean operators were applied for topic-based searches with the following keywords: (“VR” OR “Virtual Reality”) AND (“mindfulness” OR “meditation”). The search covered publications up to July 29, 2025. A total of 1,858 records were retrieved, including 1,783 in English and 75 in Chinese.

Step 2: Literature Screening.

All retrieved records were imported into EndNote for management. Titles and abstracts were carefully reviewed to identify studies that met the predefined criteria. The screening criteria were as follows: (1) The study must include actual measurement of both VR and mindfulness; (2) The study must analyze the relationship between VR and mindfulness; (3) For duplicate publications, only one version was retained. After screening, 108 studies were identified, including 102 in English and 6 in Chinese.

Step 3: Eligibility Assessment.

The full texts of the remaining studies were downloaded and reviewed to determine their eligibility for inclusion in the meta-analysis. The eligibility criteria were as follows: (1) The study was an experimental or quasi-experimental design; (2) The study included group comparisons to examine differences in mindfulness levels before and after VR interventions; and (3) Sufficient data were reported to extract effect sizes (i.e., means, standard deviations, and sample sizes). Based on these criteria, 27 studies were retained, including 24 in English and 3 in Chinese.

Step 4: Study Inclusion.

During the final inclusion stage, two studies with evident methodological or quality issues were excluded. After completing the four-step process of identification, screening, eligibility assessment, and inclusion, a total of 25 studies met the criteria for meta-analysis. Among these, 22 were published in English and 3 in Chinese, involving a combined independent sample of 1,485 participants. The included studies were conducted across seven countries: China (7 papers), Australia (6 papers), the United States (4 papers), Spain (4 papers), Slovenia (2 papers), Canada (1 papers), and Italy (1 papers).

### 3.2 Data extraction and coding

For each study, data were extracted and coded according to the following characteristics: author, year of publication, study region, sample size, mean, and standard deviation before the intervention; sample size, mean, and standard deviation after the intervention; type of experimental design; participants’ health status; duration of intervention; and measurement instruments used. The coding was completed by two coders, with an inter-coder reliability coefficient of 0.96. In cases of discrepancies, disagreements were resolved through discussion and consensus. In total, 45 effect sizes were included in the analysis.

### 3.3 Data processing and analysis

The data from the primary studies were analyzed using Comprehensive Meta-Analysis (CMA) software to calculate effect sizes and to evaluate the magnitude of the effects. In this study, Hedges’ g was selected as the effect size index. Hedges’ g is a standardized mean difference that provides a bias-corrected version of Cohen’s d, making it particularly suitable for studies with relatively small sample sizes. This correction allows for a more accurate estimation of the true effects across groups (43).

Meta-analyses can be conducted using either a fixed-effect model or a random-effects model. The fixed-effect model assumes that differences between studies are primarily attributable to sampling error, whereas the random-effects model assumes that between-study differences may arise not only from sampling error but also from variations in study populations, measurement instruments, and other study-specific factors. The choice between fixed-effect and random-effects models can be guided by the Q statistic and the I^2^ index. An I^2^ value exceeding 75% indicates high heterogeneity among studies, suggesting that a random-effects model should be employed (44).

In the present study (see Table 1), the heterogeneity test for the effect of VR on mindfulness yielded Q = 214.334 (*p* < 0.001) and I^2^ = 79.471%, indicating that 79.471% of the variance in the model can be attributed to between-study differences. Since the heterogeneity exceeds 75%, the included studies exhibit high heterogeneity, and a random-effects model was therefore selected for the main effect analysis.

**Table 1:** Overall effect, model selection and heterogeneity tests.

Model of effects	Efficacy(g)	Number of effects	95%CI	Heterogeneity tests				
				Q	df	Z	*p*	I^2^
Fixed effect model	0.949	45	[0.872,1.026]	214.334	44	24.111	0.000	79.471
Random effect model	0.975	45	[0.792,1.157]	214.334	44	10.470	0.000	79.471

CI, confidence interval; Q, Cochran’s Q.

To further explore possible moderating factors, this study conducted a subgroup analysis to examine the differences in effect sizes between different groups. The intergroup differences were determined by the Q-value statistic and its significance level.

### 3.4 Assessment of publication bias

Publication bias refers to the tendency for studies with significant results to be more likely published, which may lead to a meta-analysis overlooking other relevant studies and, consequently, result in an incomplete representation of all research conducted in the field (45). To assess the presence of publication bias in the included sample, multiple methods were employed, including a funnel plot, Egger’s regression test, and the Fail-Safe N.

**Figure 2:**
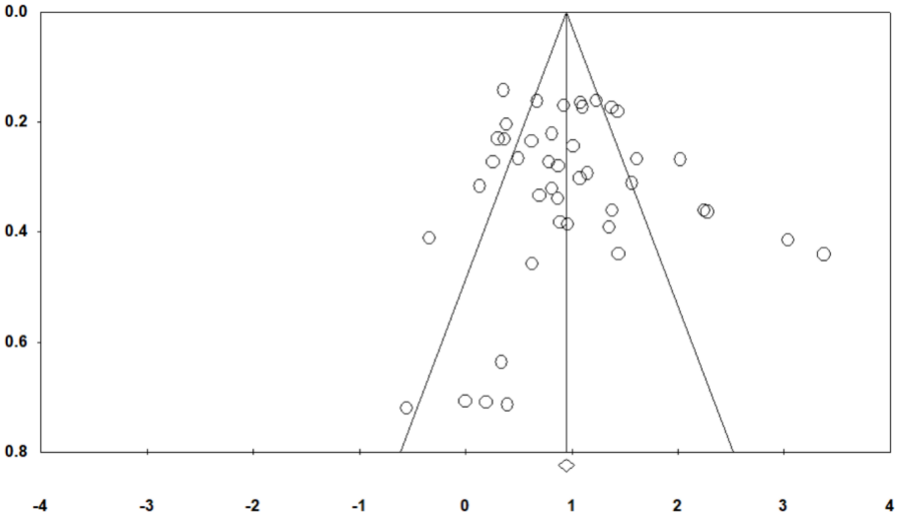
Publication bias funnel plot.

First, the funnel plot (Figure 2) showed an approximately symmetrical distribution of effect sizes, suggesting a low risk of publication bias. Second, Egger’s linear regression test was not significant (*p* = 0.77967 > 0.05), further indicating that the sample was unlikely affected by publication bias. Finally, the Fail-Safe N was 5,833, far exceeding the criterion of 5n + 10 = 235. Based on these results, there is sufficient evidence to conclude that publication bias is unlikely in this study, and the estimated effect sizes are robust and reliable.

## 4 Results

### 4.1 Analysis of main effects

The meta-analysis results (see Table 1) indicate that, under the random-effects model, the overall effect size was *g* = 0.975 (95% CI [0.792, 1.157], Z = 10.470, *p* < 0.001). This suggests that mindfulness levels after VR interventions were significantly higher than those before the intervention. According to Borenstein et al., effect sizes can be interpreted as follows: <0.2 = small effect, 0.2–0.5= small-to-moderate effect, 0.5 = moderate effect, 0.5–0.8 = moderate-to-large effect, and ≥0.8 = large effect (46). Based on this criterion, the effect of VR on enhancing mindfulness can be considered large. Mindfulness training through VR shows significant improvement.

### 4.2 Analysis of moderating effects

The heterogeneity test showed that a high degree of heterogeneity among the studies. In order to explore the source of heterogeneity, the study conducted a moderating effect test, and the specific results are shown in Table 2. The data showed that:

**Table 2:** Moderating effect test.

Moderating effect	Group	Effect size	Effect value	95% Confidence interval		Two-tailed test		Heterogeneity test	
				Lower limit	Upper limit	Z value	*p*-value	Q	*p*-value
Mode of use	Fully immersive	26	1.219	0.976	1.461	9.863	0.000	14.786	0.000
	Active interactive mode	19	0.610	0.416	0.804	6.166	0.000		
Health status	Healthy	22	0.824	0.581	1.066	6.658	0.000	9.639	0.008
	Chronic disease	4	0.236	−0.439	0.912	6.686	0.493		
	Mental disorder	19	1.225	0.966	1.483	9.281	0.000		
Study design	Randomized trial	28	1.075	0.838	1.311	8.892	0.000	2.234	0.135
	Non-randomized trial	17	0.810	0.556	1.064	6.257	0.000		
Intervention duration	Single-session intervention	19	1.134	0.777	1.490	6.233	0.000	1.268	0.260
	Multi-week intervention	26	0.900	0.705	1.095	9.050	0.000		
Measurement tool	FFMQ	7	1.157	0.634	1.681	4.334	0.000	5.672	0.225
	MAAS	20	0.793	0.606	0.981	8.299	0.000		
	SMS	5	0.967	0.438	1.486	3.585	0.000		
	TMS	9	1.515	0.802	2.229	4.161	0.000		
	Other	4	0.713	0.325	1.101	3.598	0.000		

The moderating effect of usage mode was significant. Subgroup analysis indicated that the effect of VR on enhancing mindfulness varied depending on the usage mode (Q = 14.786, *p* < 0.05). The fully immersive group showed a higher effect size (*g* = 1.219) compared to the active-interaction group (*g* = 0.610), suggesting that, relative to an active-interaction mode, a fully immersive usage mode produces a stronger enhancement of mindfulness levels.

The moderating effect of participants’ health status was significant. Subgroup analysis revealed that the effect of VR on enhancing mindfulness differed significantly across health-status groups (Q = 9.639, *p* = 0.008 < 0.05). Specifically, individuals with mental disorders showed the largest and significant intervention effect (*g* = 1.225, 95% CI [0.966, 1.483], *p* < 0.001), healthy individuals also exhibited a significant effect (*g* = 0.824, 95% CI [0.581, 1.066], *p* < 0.001), whereas the effect in individuals with chronic illnesses was not significant (*g* = 0.236, 95% CI [−0.439, 0.912], *p* = 0.493 > 0.05).

The moderating effect of study design was not significant. Although both randomized experiments (*g* = 1.075, 95% CI [0.838, 1.311], *p* < 0.001) and non-randomized experiments (*g* = 0.810, 95% CI [0.556, 1.064], *p* < 0.001) showed significant effects, there was no significant difference between the two groups (Q = 2.234, *p* = 0.135).

The moderating effect of intervention duration was not significant. Although both single-session training (*g* = 1.134, 95% CI [0.777, 1.490], *p* < 0.001) and multi-week programs (*g* = 0.900, 95% CI [0.705, 1.095], *p* < 0.001) showed significant effects, the difference between the groups was not statistically significant (Q = 1.268, *p* = 0.260 > 0.05).

The moderating effect of measurement instruments was not significant. Significant effects were observed across different scales, including the FFMQ (*g* = 1.157, 95% CI [0.634, 1.681], *p* < 0.001), MAAS (*g* = 0.793, 95% CI [0.606, 0.981], *p* < 0.001), SMS (*g* = 0.967, 95% CI [0.438, 1.486], *p* < 0.001), TMS (*g* = 1.515, 95% CI [0.802, 2.229], *p* < 0.001), and other scales (*g* = 0.713, 95% CI [0.325, 1.101], *p* < 0.001). However, the differences between these groups were not statistically significant (Q = 5.672, *p* = 0.225 > 0.05).

## 5 Discussion

### 5.1 Effectiveness of VR in enhancing mindfulness

Over the past four decades, the application of mindfulness has expanded considerably, with mindfulness training increasingly recognized as an effective approach to alleviating disease-related distress and improving quality of life (47). With the rapid advancement of information technologies, growing attention has been directed toward leveraging digital media to facilitate mindfulness training, aiming to overcome the limitations of traditional approaches and to enhance training effectiveness (48). However, few researchers have considered the intervention effect of VR as a whole. This study adopted the method of meta-analysis, and included 25 Chinese and English studies, 45 effect sizes and 1,485 participants for systematic integration. The results revealed that, under the random-effects model, VR had a significant positive effect on mindfulness, with an overall effect size of *g* = 0.975 (95% CI [0.792, 1.157], *p* < 0.001). According to the classification criteria proposed by (46), this represents a large effect. These findings suggest that VR technology has a substantial impact on enhancing mindfulness and provide strong empirical support for the application of VR in mindfulness-based interventions.

In traditional mindfulness training, practitioners are required to sustain a high level of concentration, yet the environments in which they practice are often vulnerable to external distractions. For beginners in particular, this can lead to increased susceptibility to mind-wandering or difficulties in maintaining patience (49). VR technology can effectively overcome these limitations. Its unique advantage lies in the ability to construct immersive, engaging, and controllable environments (50). Such environments not only enhance users’ cognitive and emotional trust in VR and foster deeper emotional connections, but also suppress perceptions of external risks. Moreover, at the behavioral level, VR environments promote continuous acceptance and adoption, thereby facilitating the formation, development, and maintenance of novel human–machine relationships (51).

Attention Restoration Theory (ART) further provides a theoretical foundation for VR-based mindfulness training. This theory emphasizes the positive role of natural environments in restoring individuals’ attentional resources, effectively enhancing focus and cognitive function. The key advantage of VR lies in its ability to create realistic, immersive virtual environments that simulate nature. Such environments allow participants to disconnect from stressors and distractions, enabling them to focus their attention on present-moment awareness (22). For example, when users are immersed in a simulated forest or beach scene, elements within the environment—such as natural sounds and the harmony of the landscape—can serve to restore attention, thereby enhancing focus during meditation. Whether for beginners or experienced practitioners, VR-based mindfulness interventions can improve their state of mindfulness (52).

### 5.2 Moderating effect analysis

#### 5.2.1 Usage mode: fully immersive mode is better than active interaction mode

Subgroup analysis revealed a significant moderating effect of interaction mode on the effectiveness of VR interventions (Q = 14.786, *p* < 0.001). Specifically, the fully immersive mode (*g* = 1.219) demonstrated a significantly stronger effect than the active-interactive mode (*g* = 0.610), thereby supporting Hypothesis 1. This finding may be explained by the role of cognitive load in mindfulness training. Cognitive load theory posits that cognitive load during learning is composed of intrinsic cognitive load, extraneous cognitive load, and germane cognitive load. During the learning process, if learners lack sufficient prior knowledge or if the presentation of content and materials is overly complex, it is likely to impose significant cognitive load on the learner. In brief, the core idea of cognitive load theory is that human working memory capacity is limited, and effective learning and information processing require avoiding excessive cognitive load (53). Within VR interventions, participants in fully immersive settings follow relatively simple operational procedures: they primarily attend to the audio guidance of a virtual instructor and immerse themselves in the virtual environment. This process facilitates attentional anchoring rather than frequent switching, the cognitive load on learners is relatively low, thereby enhancing present-moment awareness. For beginners, such straightforward engagement also helps mitigate potential anxiety about technology. In contrast, the active interactive mode requires participants to allocate attentional resources to managing hand controllers, navigating between scenes, and performing multiple tasks simultaneously. These additional demands can interfere with perceptual awareness and hinder sustained focus on breathing and bodily sensations, the cognitive load imposed on learners is considerable. Such divided attention runs counter to the core principle of mindfulness practice, which emphasizes non-judgmental and effortless awareness of the present moment.

#### 5.2.2 Health status: individuals with mental disorders benefit the most, while effects are limited for those with chronic diseases

The moderating effect of health status was significant (Q = 9.639, *p* = 0.008), assumption 2 was verified. VR interventions yielded the greatest benefits for individuals with mental disorders such as anxiety and depression (*g* = 1.225, 95% CI [0.966, 1.483], *p* < 0.001). Anxiety and depression are frequently associated with deficits in attentional control and tendencies toward negative rumination (54). According to stress reduction theory, exposure to natural environments promotes relaxation, calmness, and reflection, thereby mitigating attentional control deficits and reducing the negative impact of maladaptive rumination (55). Compared with mindfulness training conducted in urban settings, nature-based mindfulness practices have been shown to produce more pronounced improvements in mood and psychological well-being (56). VR can simulate natural environments such as rivers, oceans, flowers, and forests, thereby reducing the influence of real-world stressors. At the same time, it enables participants to transform abstract negative emotions into concrete and observable experiences, enhancing their capacity for emotional acceptance (57).

The effect size for healthy individuals was also found to be high (*g* = 0.824, 95% CI [0.581, 1.066], *p* < 0.001), exceeding the impact reported for traditional mindfulness interventions in this population. A meta-analysis revealed that the effect of traditional mindfulness on healthy people was Hedges’ g = 0.55 (58). This analysis, which integrated data from 29 studies and 2,668 participants, found that traditional mindfulness had a moderate effect on stress, anxiety, depression and quality of life, and that this effect remained after several weeks of follow-up (58). More recently, emerging VR-based mindfulness interventions have shown even greater benefits for healthy populations. For example, one study found that VR meditation not only made mindfulness practice more engaging but also significantly outperformed audio-guided meditation in promoting relaxation, enhancing positive affect, and reducing stress (59). These findings further support the notion that VR can serve as an effective tool for improving quality of life and subjective well-being among healthy individuals.

For individuals with chronic illnesses, the effect size was not significant (*g* = 0.236, 95% CI [−0.439, 0.912], *p* = 0.493). Although many studies have suggested that VR can alleviate negative emotions among patients with chronic conditions, consensus regarding its therapeutic efficacy in this population has yet to be established. Some scholars argue that VR technology has shown limited effectiveness in chronic pain management (60). They contend that many existing findings were derived from small-scale experimental studies, and that systematic reviews specifically addressing VR-based mindfulness interventions for chronic conditions remain lacking, requiring further support from high-quality research (61). The present study found the limited effectiveness of VR for individuals with chronic illnesses. However, this finding should be interpreted with caution, as only four studies with a total sample size of 17 participants were included in this meta-analysis. Future research should incorporate larger and more representative samples to validate these results.

#### 5.2.3 Study design: no significant difference between randomized and non-randomized trials

The moderating effect of study design was not significant (Q = 2.234, *p* = 0.135), and Hypothesis 3 was not supported. This indicates that the results of randomized and non-randomized experiments showed convergence in current research, with both types of experiments confirming the effectiveness of VR in enhancing mindfulness levels. These findings suggest that results from high-quality non-randomized studies still hold a degree of credibility. Some researchers argue that although RCTs are considered the gold standard for causal inference, well-conducted non-randomized studies can also provide reliable evidence, particularly in intervention studies involving technology-assisted approaches (62). Additionally, the technological advantages of VR may help reduce variability during the intervention and mitigate potential confounding risks in non-randomized trials (63). Therefore, at the current stage, evidence from non-randomized studies should not be entirely discounted, especially in contexts where RCTs face ethical or feasibility constraints.

#### 5.2.4 Intervention duration: no significant moderating effect

The moderating effect of intervention duration was not significant (Q = 1.268, *p* = 0.260), and Hypothesis 4 was not supported. Both single-session and multi-week VR interventions were effective in enhancing mindfulness levels. This finding indicates that the effectiveness of VR interventions does not entirely depend on the length of the intervention period. For instance, a study involving healthy adults demonstrated that even a single 20-min VR mindfulness session could significantly reduce negative psychological states and improve mindfulness levels (57). These results suggest that VR-based mindfulness interventions exhibit strong flexibility and adaptability with respect to intervention duration, making them suitable for both rapid stress relief and longer-term, systematic mindfulness training.

#### 5.2.5 Measurement tools: no significant moderating effect

The moderating effect of measurement tools was not significant (Q = 5.672, *p* = 0.225), and Hypothesis 5 was not supported. This finding reflects the robustness of VR in enhancing mindfulness across different assessment instruments. Although the scales such as FMMQ, MAAS, SMS and TMS have different focus on application, the immersive environment constructed by VR can not only enhance individuals’ awareness at a specific moment to improve state mindfulness, but also help individuals develop trait mindfulness through continuous practice. These results further support VR as an effective medium for improving mindfulness across multiple dimensions. Consequently, researchers can select the most appropriate measurement tool based on the specific objectives of their study.

The above studies have demonstrated that usage mode and participants’ health status have significant moderating effects, while the moderating effects of study design, intervention duration, and measurement tools were not significant. Due to incomplete data in the original literature, this study only examines the moderating effects of the five factors mentioned above and does not investigate the moderating effects of other factors such as individual traits (e.g., technology acceptance) or VR device models. Future research should focus on exploring these additional factors.

## 6 Conclusion

(1) VR technology can significantly enhance mindfulness, and the effect is large. (2)The effectiveness of VR in improving mindfulness was moderated by both usage mode and participants’ health status. Fully immersive modes outperformed active-interactive modes, and individuals with mental disorders derived greater benefits more than healthy individuals, whereas the intervention effect for individuals with chronic illnesses was not significant. (3)The moderating effects of study design, intervention duration, and measurement tools were not significant.

The findings of this study enrich the theoretical foundation of mindfulness training and provide clear guidance for future VR intervention design. Specifically, VR interventions should prioritize constructing highly immersive, low-interaction-complexity virtual environments centered on natural settings, avoiding distractions caused by excessive user operations. Tailored approaches can be implemented to accommodate the diverse needs of different populations: for individuals with psychological disorders such as anxiety and depression, VR can serve as an accessible and engaging treatment modality; for healthy individuals, it can be an effective tool for daily stress management and emotional regulation; and when promoting VR among chronic illness patients, careful evaluation of its suitability is essential, potentially integrating VR with other therapeutic methods. Social organizations such as schools and corporations can introduce VR mindfulness programs to enhance students’ or employees’ focus, psychological resilience, and overall mental health. Furthermore, it is recommended that public health authorities, research institutions, and technology companies strengthen collaboration to promote the standardization and normalization of VR mindfulness initiatives, integrating them into the broader digital mental health service framework.

## 7 Limitations and future research

This study has the following limitations: First, it only included Chinese and English literature, failing to incorporate other language types, which may have resulted in some studies being overlooked. To address this language limitation, future studies could collaborate with multilingual researchers to access literature in additional languages. Second, the sample size for chronic illness population was relatively small, requiring cautious interpretation of the findings. Future research should aim to expand the sample size. Third, differences in VR hardware and software, such as headset types and scene design, were not thoroughly considered. Future studies should conduct more detailed research that includes VR device models, environmental design specifics, and other key parameters. Fourth, it is necessary to further subdivide groups, such as the older adults and college students. Age, social identity and other characteristics may affect the relationship between digital media and mindfulness level (6).

## Data Availability

The original contributions presented in the study are included in the article/supplementary material, further inquiries can be directed to the corresponding author.
